# Pharmacologic control of oxidative stress and inflammation determines whether diabetic glomerulosclerosis progresses or decreases: A pilot study in sclerosis-prone mice

**DOI:** 10.1371/journal.pone.0204366

**Published:** 2018-09-25

**Authors:** Fabrizio Grosjean, Elena M. Yubero-Serrano, Feng Zheng, Vittoria Esposito, Shobha Swamy, Sharon J. Elliot, Weijing Cai, Helen Vlassara, Fadi Salem, Gary E. Striker

**Affiliations:** 1 Division of Nephrology, Dialysis and Transplantation, Fondazione IRCCS Policlinico San Matteo, Pavia, Italy; 2 Lipids and Atherosclerosis Unit, Instituto Maimónides de Investigación Biomédica de Córdoba (IMIBIC), Reina Sofia University Hospital, University of Cordoba, and CIBER Fisiopatologia Obesidad y Nutricion (CIBEROBN), Instituto de Salud Carlos III, Madrid, Spain; 3 Division of Nephrology and Basic Science Laboratory, Union Hospital Fujian Medical University, Fuzhou, Fujian, China; 4 Unit of Nephrology and Dialysis, Fondazione IRCCS Salvatore Maugeri, Pavia, Italy; 5 Division of Pulmonary, Allergy and Critical Care, Department of Medicine, University of Alabama School of Medicine, Birmingham, Alabama, United States of America; 6 Department of Surgery, School of Medicine, University of Miami, Miami, Florida, United States of America; 7 Division of Experimental Diabetes and Aging, Department of Geriatrics, Icahn School of Medicine at Mount Sinai, New York, New York, United States of America; 8 Department of Pathology, Icahn School of Medicine at Mount Sinai, One Gustave Levy Place, Annenberg 15–235, New York, New York, United States of America; 9 Division of Experimental Diabetes and Aging, Department of Geriatrics and Division of Nephrology, Department of Medicine, Icahn School of Medicine at Mount Sinai, New York, New York, United States of America; Hospital Universitario de la Princesa, SPAIN

## Abstract

Diabetic kidney disease (DKD) is characterized by progressive glomerulosclerosis (GS). ROP mice have a sclerosis-prone phenotype. However, they develop severe, rapidly progressive GS when rendered diabetic. Since GS also develops in aged C57Bl6 mice, and can be reversed using bone marrow from young mice which have lower oxidative stress and inflammation (OS/Infl), we postulated that this might also apply to DKD. Therefore, this pilot study asked whether reducing OS/Infl in young adult sclerosis-prone (ROP) diabetic mice leads to resolution of existing GS in early DKD using safe, FDA-approved drugs.After 4 weeks of stable streptozotocin-induced hyperglycemia 8–12 week-old female mice were randomized and treated for 22 weeks as follows: 1) enalapril (EN) (n = 8); 2) pyridoxamine (PYR)+EN (n = 8); 3) pentosan polysulfate (PPS)+EN (n = 7) and 4) PPS+PYR+EN (n = 7). Controls were untreated (non-DB, n = 7) and hyperglycemic (DB, n = 8) littermates. PPS+PYR+EN reduced albuminuria and reversed GS in DB. Treatment effects: 1) Anti-OS/Infl defenses: a) PPS+PYR+EN increased the levels of SIRT1, *Nrf2*, estrogen receptor α (ER*α*) and advanced glycation endproduct-receptor1 (AGER1) levels; and b) PYR+EN increased ER*α* and AGER1 levels. 2) Pro-OS/Infl factors: a) PPS+PYR+EN reduced sTNFR1, b) all except EN reduced *MCP1*, c) RAGE was reduced by all treatments. In summary, PYR+PPS+EN modulated GS in sclerosis-prone hyperglycemic mice. PYR+PPS+EN also decreased albuminuria, OS/Infl and the sclerosis-prone phenotype. Thus, reducing OS/Infl may reverse GS in early diabetes in patients, and albuminuria may allow early detection of the sclerosis-prone phenotype.

## Introduction

While the pathogenesis and causes of glomerulosclerosis (GS) are unclear, it has been thought to be inevitably progressive. Diabetic nephropathy (DKD), the most common form of GS in adults, is restricted to a subset of diabetic patients (~20–40%) suggesting a role for a sclerosis-prone genetic background, similar to that described in animal models [1–3]. Factors in addition to hyperglycemia [4] and genetic susceptibility [5] that may influence the development of DKD include gender, age and dietary intake [6–8].

The first successful attempt to identify components of the sclerosis-prone phenotype was a comparison of young adult ROP Os+ (sclerosis-prone) and C57 Os/+ (sclerosis-resistant) mice by transcriptomes [6]. This study “highlighted the roles for oxidative stress” and “provided evidence for activation of the pro-fibrotic and pro-inflammatory transforming growth factor β1 (TGFβ1) signal” in the genetic susceptibility to GS. These data complemented other data suggesting that the pathogenesis of DKD includes both environmental [9–11] and genetic factors [5, 12] in which inflammation and oxidative stress (OS/Infl) may play pivotal roles [6, 13–17].

The modern human diet and most pelleted experimental animal food contain a ubiquitous group of environmental factors, including glycation endproducts (AGEs), which are strong inducers of OS/Infl and are a risk factor for DKD and chronic kidney disease (CKD) [7, 8, 10, 11, 16, 17]. We found that GS develops as OS/Infl increases in normal aging, and that GS can be reduced by controlling the oral intake of agents that increase OS/Infl, such as AGEs [18].

Additionally, GS can be reduced by the transplantation of bone marrow from young mice, which have intact OS/Infl defenses, into old mice, which have lowered OS/Infl defenses [19, 20]. In addition GS is induced in young mice after administration of bone marrow from old mice.

These studies support a role for OS/Infl in the development as well as the regression of GS independently of hyperglycemia. However, the effect of OS/Infl on the sclerosis-prone phenotype and to gender has not been further tested.

A search for drugs that control OS/Infl revealed that pentosan polysulfate (PPS) blocks certain cell surface pro-inflammatory receptors, such as tumor necrosis factor- α (TNF-α). In addition, pyridoxamine (PYR) blocks intracellular pro-OS/Infl factors, such as methylglyoxal (a very reactive AGE) [21, 22]. Recently, we found that PYR treatment of anestrus (21 months-old) female C57Bl6 mice resulted in increased renal expression of anti-oxidant defenses such as estrogen receptor α (ERα), Sirtuin 1 (SIRT1) and AGER1 (a receptor for AGEs), while decreasing TGFβ1 in aged mice [23]. We postulated that the combination of enalapril (EN), PPS and PYR might provide protection against the development of GS in hyperglycemic sclerosis-prone mice. In this pilot study we chose young hyperglycemic adult, female ROP Os/+ mice at an age at which they were known to developed, progressive GS and albuminuria, but not renal failure [2, 24].

Our aim was to determine if reducing OS/Infl modulated established GS and modified sclerosis-prone phenotype using combinations of FDA-approved generic drugs (PPS, PYR and EN).

## Materials and methods

### Experimental design

Female ROP Os/+mice were obtained from Jackson Laboratories (Bar Harbor, ME, USA). The protocol was approved by the Icahn School of Medicine at Mount Sinai Institutional Animal Care and Use Committee (Protocol # 080328). Animals were housed in groups of two to four in plastic cages with corncob bedding, maintained on a 12-hour light:dark cycle, and provided with standard laboratory animal chow and water *ad lib*. Stable hyperglycemia (DB) was induced at 8–12 weeks of age by 7 to 14 intraperitoneal injections of STZ(50 μg/g body wt), or vehicle (citrate buffer) as a control, administered within a two week period [24]. Mice were fed with standard laboratory animal chow. While we have found that standard laboratory contain large amounts of AGEs, we decided to utilize this chow so that the results of these experiments could be more easily reproduced in other laboratories [25]. Mice were weighed and fasting blood glucose levels were determined by tail vein nicking at baseline and weekly thereafter. After 4 weeks of stable hyperglycemia (>200 mg/dl), mice (DB) were randomized to the following treatment groups: EN (10 mg/kg/day, n = 8), PYR+EN (PYR: 200mg/kg/day, n = 8), PPS+EN (PPS: 25mg/kg/day, n = 7), and PPS+PYR+EN (n = 7). Untreated non-DB (non-DB, n = 7) and untreated DB mice (DB, n = 8) served as controls. The estimated number of animals was based on our prior laboratory experience in this ROP/Os + mouse strain, with and without the induction of hyperglycemia [26, 27].

Intraperitoneal NPH insulin was given to maintain blood glucose levels below 300 mg/dl. EN was given to mimic the standard treatment now extant for patients with DKD [28]. Urine albumin excretion was measured weekly by ELISA (Bethyl Laboratories Inc., Houston, TX) [29]. Urine creatinine levels were measured by a colorimetric assay (Cayman Chemical Company, Ann Arbor, MI), and the urine albumin excretion rate was expressed as the ratio of albumin to creatinine (ACR) [9, 29]. Blood urea nitrogen (BUN) levels were evaluated by a colorimetric method (TECO Diagnostics, California, USA) [29, 30]. After 22 weeks of follow-up, mice were terminally anesthetized with ketamine/xylazine according to Guidelines established by the National Institutes of Health (NIH). Level of anesthesia was assessed by pedal reflex and all efforts were made to minimize suffering.

The study was reviewed and approved by the Institutional Animal Care and Use Committee of the Icahn School of Medicine at Mount Sinai, as a prerequisite for submission of the original grant [Juvenile Diabetes Research Foundation (Grant number 17-2008-1041)], which supported this pilot study.

### Histology

Kidneys were flushed with phosphate-buffered saline and then fixed in 4% paraformaldehyde [9, 29]. Forty eight hours after fixation, tissue samples were embedded in glycol methacrylate and 4μm sections were stained with periodic acid Schiff.

### Glomerular morphometry

Morphologic analysis was performed in periodic acid–Schiff (PAS) stained sections [31]. A total of 30 glomeruli were randomly selected from each kidney by moving the slide from the outer to the inner cortex in a random fashion to obtain non-overlapping sample fields. Glomerular images were recorded using a CCD camera (Sony, Tokyo, Japan) mounted on a light microscope (Zeiss, Gottingen, Germany). Glomerular tuft surface area was obtained using the MetaMorph image analysis computer program (Universal Imaging Co., West Chester, PA). From the glomerular tuft image, the amount of PAS–positive material was selected automatically by use of the color recognition properties of the software. The number of pixels in the PAS-positive area was considered to represent the area of sclerosis and was expressed as a fraction of the tuft surface area [30].

### Plasma sTNFR1

Plasma soluble tumor necrosis factor receptor-1 (sTNFR1) levels were determined by ELISA kits following the manufacturers’ protocols (eBioscience, Inc. San Diego, Ca, USA and R&D Systems Inc., Minneapolis, MN, USA respectively).

### Circulating and tissue AGEs

The amount of AGEs in the tissue (liver), and plasma was determined by a competitive enzyme-linked immunosorbent assay using a monoclonal antibody reacting with *N*-carboxymethyl-lysine (CML, 4G9 mAb) or Methylglyoxal (MG, 3D11 mAb) [25]. Tissue values were corrected to the protein sample concentrations. *N*-carboxymethyl-lysine-bovine or Methylglyoxal serum albumin were used as standards for quantitation.

### Oxyblot

An oxyblot protein oxidation detection kit (Chemicon International, Temecula, CA) was used to assess overall carbonyl groups introduced into the protein side chain by oxidative modification in renal tissues [9]. 2,4-Dinitrophenylhydrazine derivatization was performed for 15 minutes following the manufacturer's instructions on total 10 μg of protein obtained from the kidney tissue lysate. The dinitrophenylhydrazine-derivatized protein samples were separated by 12%SDS-polyacrylamide gel electrophoresis. Proteins were transferred to polyvinylidenedifluoride membranes, stained by Ponceau Red, and then probed with an anti-dinitrophenylhydrazine antibody. Blots were developed using a chemiluminescence detection system. No visible bands were seen in samples without reaction with 2,4-dinitrophenylhydrazine before Western blots (data not shown).

### Real time PCR studies

Total RNA was isolated from the renal cortex using a PureYield RNA Midiprep kit (Promega, Madison, WI) and the preparation was freed of DNA contamination by incubating with DNase I. Equal amounts of RNA from the samples were then reverse-transcribed as described previously [32]. The mRNA levels of *TGFβ1*, a receptor for AGEs (*RAGE*), monocyte chemotactic protein-1 (*MCP1*), nuclear factor (erythroid-derived 2)-like 2 (*Nrf2*) and estrogen receptor-α (*ERα*) were determined by real-time PCR under standard conditions using the SYBR Green PCR Master Mix (Applied Biosystems; Warrington, UK). The primer sequences were: *TGFβ1*, forward, 5’-GTGCGGCAGCTGTACATTGACTTT and reverse, 5’-TGTACTGTGTGTCCAGGCTCCAAA; *RAGE*, forward 5’-CCTGGGAAGCCAGAAATT and reverse 5’-ACACAGGTCAAGGTCACA; *MCP1*, forward, 5’-AGGTCCCTGTCATGCTTCTC and reverse, 5’-TCATTGGGATCATCTTGCT: *Nrf2*, forward, 5’-TCCATTCCCGAATTACAGTGTC and reverse, 5’-CACAGTTGCCCACTTCTTTTT; *ERα*, forward, 5’-ATTGGTCTCGTCTGGCGCT and reverse, 5’-CTCCACCATGCCCTCTACACA. The mRNA levels in each sample were normalized by *β-actin* mRNA levels [9].

### Western blots

Proteins were isolated from the renal cortex after homogenization and sonication in a lysis buffer containing protease and phosphatase inhibitors at 4° C (Pierce, Rockford, IL). Samples were centrifuged at 4° C at 14,000 rpm for 15 minutes, the supernatant was recovered and the protein concentration was measured by a colorimetric assay according to the manufacturer’s instructions (BCA Protein Assay Kit, Pierce). The same amount of protein obtained from three mice was pooled from each group and a total of fifteen micrograms of protein were loaded onto 8 or 10% SDS-PAGE gels. After separation, proteins were transferred to PVDF membranes. For Western blots, membranes were pretreated with a blocking buffer (Thermo Fisher Scientific, Waltham, MA) at room temperature for 30 min before incubating with the following antibodies with appropriate dilution: anti-OST48 (AGER1) (Santa Cruz Biotechnology, Santa Cruz, CA, USA) and anti-SIRT1 (Santa Cruz). After overnight incubation with primary antibody at 4°C, membranes were washed and incubated with a horseradish peroxidase-labeled secondary antibody and immunoreactivity was detected using the enhanced chemiluminescence assay (GE Healthcare, Buckinghamshire, UK). Membranes were then washed 30 min with a stripping buffer (Thermo Fisher Scientific) and re-probed with an anti-β-actin antibody (1:5000; Sigma, St. Louis, MO) to ensure that protein loading was similar for each sample. These experiments were at least repeated twice [25].

### Statistical analysis

Data were expressed as mean value ± SE. Differences in mean were analyzed by ANOVA and a p value of less than 0.05 was considered significant. Graph Pad Prism 6.0 software was used for data analysis. Outliers were removed from the data set according Moore et al [33]. Backward multiple linear regression analysis were performed to estimate the independent contribution of blood glucose, serum and intracellular CML (sCML and iCML, respectively) and MG (sMG and iMG, respectively) levels, sTNRF1, AGER1 and SIRT-1 protein levels and *TGF*β*1*, *MCP1*, *RAGE*, *Nrf2* and *ERα* mRNA levels to ACR and GS, independently, both considered as clinical markers in the prediction of both the presence and the progression of DKD.

## Results

### General characteristics

Insulin was used to control hyperglycemia (mean = 265±81mg/dl). Blood glucose area under the curve (AUC), calculated after randomization and weekly until sacrifice, was significantly higher in all diabetic groups compared to non-DB controls (p<0.001) but was similar in all diabetic groups (**Table 1).** Body weight was significantly reduced only in the PPS+EN group compared to non-DB mice (<10%) (*p*<0.05). BUN remained in the normal range in all groups and kidney weight (unlike that found in sclerosis-resistant strains) was not altered by diabetes or treatment.

**Table 1:** Characteristics at sacrifice.

Treatment	Body weight (gr)	Kidney weight/ body weight	Blood glucose (mg/dl)	Blood glucose AUC	BUN (mg/dl)
Non-DB (n 7)	23.7 ±0.4	0.0051±0.0004	124.8±4.0	7022±202	26.22±1.36
DB (n 8)	21.8±0.8	0.0047±0.0002	289.7±37.2 ***	40318±4787***	25.74±2.36
DB EN (n 8)	21.9 ±0.6	0.0042±0.0001	269.7±30.2 **	38230±4047***	24.66±1.32
DB EN+PYR (n 8)	22.8±0.7	0.0043±0.0001	224.5±18.3 *	32556±2144***	28.19±2.06
DB EN+PPS (n 7)	20.9±0.5*	0.0044±0.0002	253.9±23.9**	37079±3493***	32.09±2.76
DB EN+PYR+PPS (n 7)	21.4±0.6	0.0044±0.0003	234.4±24.7 *	33298±2824***	30.08±2.31

BUN: blood urea nitrogen; AUC: area under the curve.

Data are expressed as mean value ± SE.

*p<0.05

**p<0.01

***p<0.001 vs. Non-DB

### Glomerulosclerosis and albuminuria

**Fig 1:**
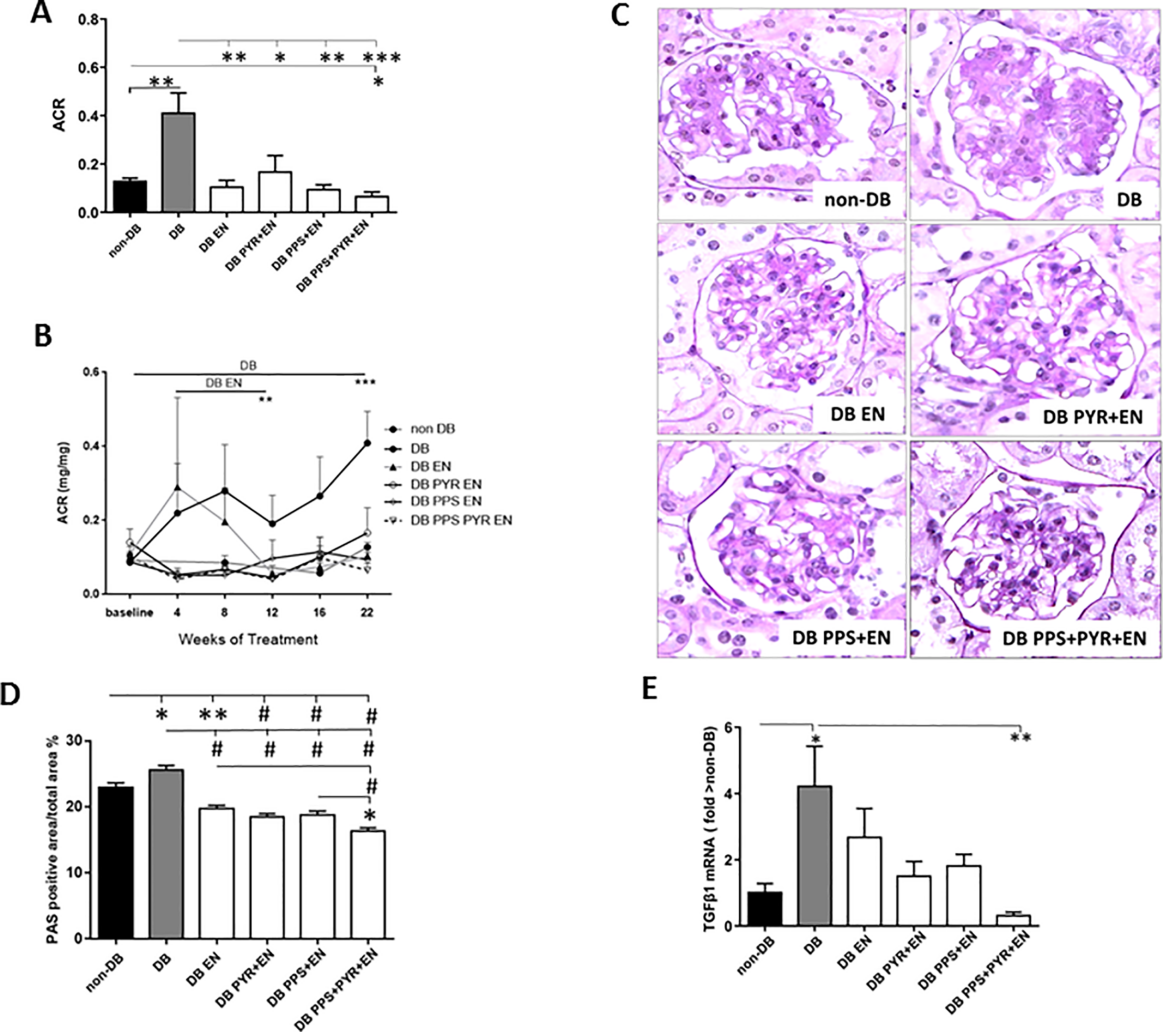
A) Albumin to creatinine ratio (ACR) at sacrifice (22 weeks). B) ACR follow-up for 22 weeks. C) Periodic acid–Schiff (PAS) staining: a) non-DB, b) DB, c) DB EN, d) DB PYR+EN, e) DB PPS+EN, f) DB PPS+PYR+EN. D) Morphometric evaluation of glomerular PAS positive areas. E) Kidney TGFβ1 mRNA levels. *p<0.05; **p<0.01; ***p<0.005; ^#^*p*<0.001. DB, diabetics; EN, enalapril; PPS, pentosan polysulfate; PYR, pyridoxamine; PAS, periodic acid–Schiff; TGFβ1, transforming growth factor β1.

ROP Os/+ mice spontaneously develop proteinuria, glomerulosclerosis, glomerular hypertrophy and are prone to develop slowly progressive DKD [24]. ACR remained stable in non-DB mice, continued to increase in DB mice to reach nearly two-fold higher levels than non-DB at 22 weeks, but was reduced in all treatment groups (**Fig 1A and 1B**). ACR was most reduced by PPS+PYR+EN (*p*<0.005) (**Fig 1A**). PPS+PYR and PPS+PYR+EN treatment reduced albuminuria below DB at 4 weeks (*p*<0.05 and *p*<0.001, respectively), an effect that continued to study end. While ACR in EN treated mice was increased at 4 weeks, it returned to non-DB levels by 12 weeks. PPS+PYR+EN reduced ACR below non-DB controls at 22 weeks (*p*<0.05) **(Fig 1A)**.

GS, as evaluated by PAS staining (**Fig 1C**), was evident in non-DB littermates and increased in DB (p<0.05), and was improved by all treatments (*p*<0.001) (**Fig 1C and 1D**). GS was more reduced by PPS+PYR+EN than by EN (*p*<0.001), or DB PPS+EN (*p*<0.05). The treatment regimens reduced GS below that in non-DB (EN *p*<0.01; PYR+EN, PPS+EN, PPS+PYR+EN p<0.001) (**Fig 1C and 1D**). None of the DB mice had tubulo-interstitial inflammatory cell infiltrates or glomerular hypertrophy compared to non-DB, consistent with previous data on ROP Os+ mice at this age [2, 3].

The increased renal *TGFβ1* mRNA levels, often associated with GS[34], were increased in DB, compared to non-DB (*p*<0.05) and reduced only by PPS+PYR+EN (*p*<0.01) (**Fig 1E**).

### Oxidative stress and inflammation

#### Pro-OS/Infl factors

**Fig 2:**
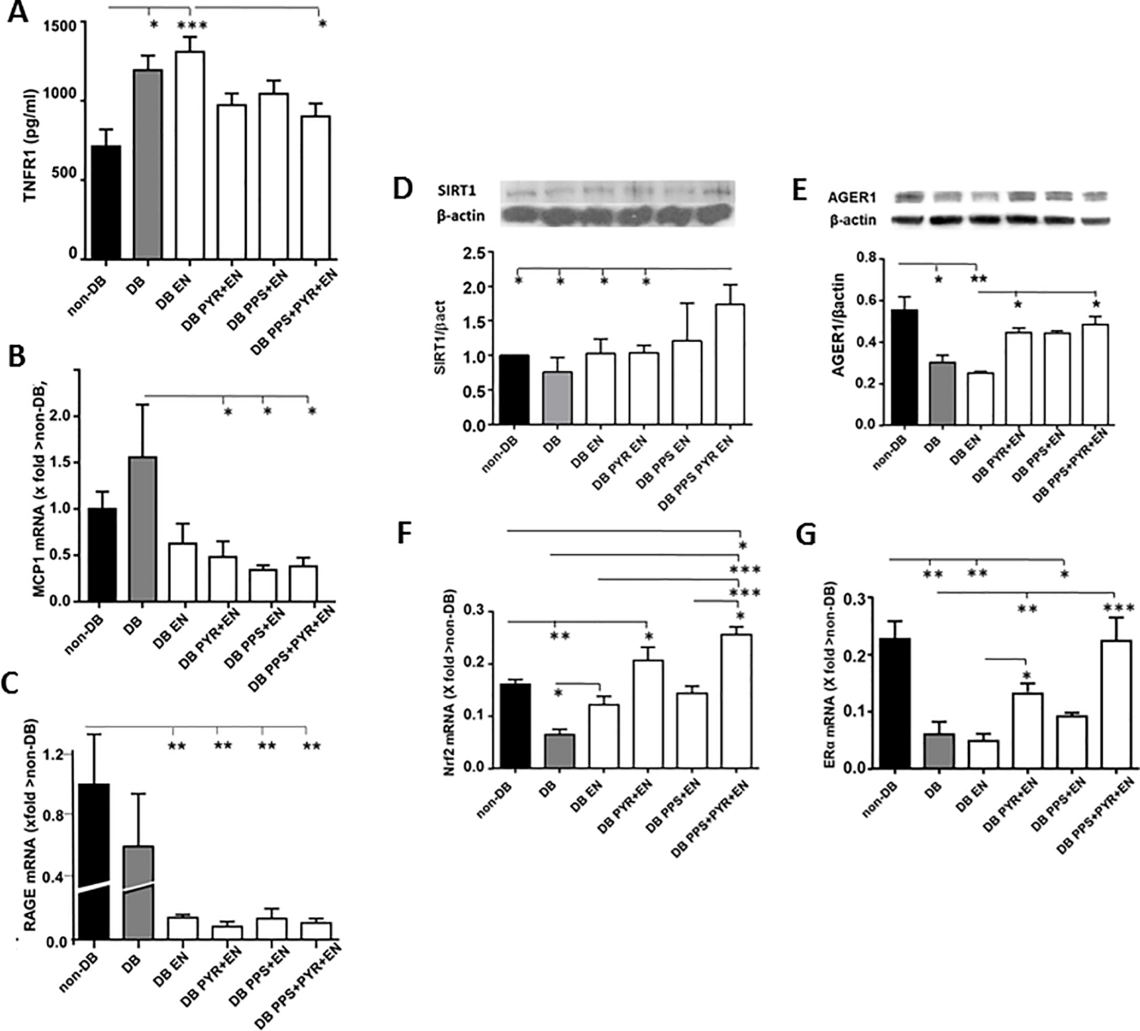
A) Plasma sTNFR1 levels. B) Kidney MCP1 mRNA levels. C) Kidney RAGE mRNA levels. D) Kidney SIRT1 protein levels. E) Kidney AGER1 protein levels. F) Kidney Nrf2 mRNA levels G) Kidney ERα mRNA levels. *p<0.05; **p<0.01; ***p<0.005; ^#^*p*<0.001. DB, diabetics; EN, enalapril; PPS, pentosan polysulfate; PYR, pyridoxamine; sTNFR1, tumor necrosis factor receptor-1; MCP1, monocyte chemotactic protein-1; RAGE and AGER1, receptors for AGEs; SIRT1, Sirtuin-1; Nrf2, nuclear factor (erythroid-derived 2)-like 2; ER*α*, Estrogen receptor *α*.

In comparison to non-DB, sTNFR1 levels were higher in DB and DB EN (*p*<0.05 and *p*<0.005, respectively) (**Fig 2A**). PPS+PYR+EN was the only treatment that significantly reduced sTNFR1 levels in DB mice (*p*<0.05), while there was only a trend in DB PYR+EN and DB PPS+EN.

*MCP1* mRNA levels [35] were not different between DB and non-DB (**Fig 2B**), but were significantly reduced in all treatment groups compared to DB (*p*<0.05) except in DB EN in which only a trend was present. There were no differences between the treatment groups and non-DB.

*RAGE* mRNA levels were not different between DB and non-DB, but were decreased in all treatment groups (*p*<0.01) (**Fig 2C)**.

#### Anti-Os/Infl factors

SIRT1 protein levels were increased in DB PPS+PYR+EN, compared to both non-DB and all DB groups except PPS+EN treated animals (*p*<0.05) (**Fig 2D**).

AGER1 protein levels were reduced in both DB (*p*<0.05) and DB+EN compared to non-DB (*p*<0.01), but were increased to approximate non-DB levels in DB mice treated with either DB PYR+EN or DB PPS+PYR+EN (*p*<0.05) (**Fig 2E**). The levels in DB PYR+EN and DB PPS+PYR+EN were higher than DB EN (p<0.05)

*Nrf2* mRNA levels were higher in non-DB mice compared to DB (*p*<0.01). *Nrf2* mRNA was increased in DB+EN compared to DB (*p*<0.05) (**Fig 2F**). *Nrf2* mRNA levels were also increased in DB PPS+PYR+EN compared to DB PPS+EN (*p*<0.05), DB EN (p<0.005), DB (p<0.005) and non-DB (p<0.05).

*ERα* mRNA levels were lower in DB compared to non-DB (*p*<0.01), DB+EN (*p*<0.01), DB PYR+EN and DB PPS+EN (*p*<0.05). *ERα* expression was also higher in DB+EN compared to DB EN+PYR (p< 0.05). However, *ERα* expression was increased by PYR+EN compared to DB (*p*<0.01). *ERα* expression was returned to non-DB control levels by PPS+PYR+EN (*p*<0.005) **(Fig 2G).**

There were no significant differences in liver tissue (intracellular) or circulating (serum) levels of CML or MG (data not shown). As in most previous studies of ROP mice at this “young/adult” age we found little histological evidence of interstitial inflammatory cells [2, 3, 24, 36].

### Oxidized total kidney protein

**Fig 3:**
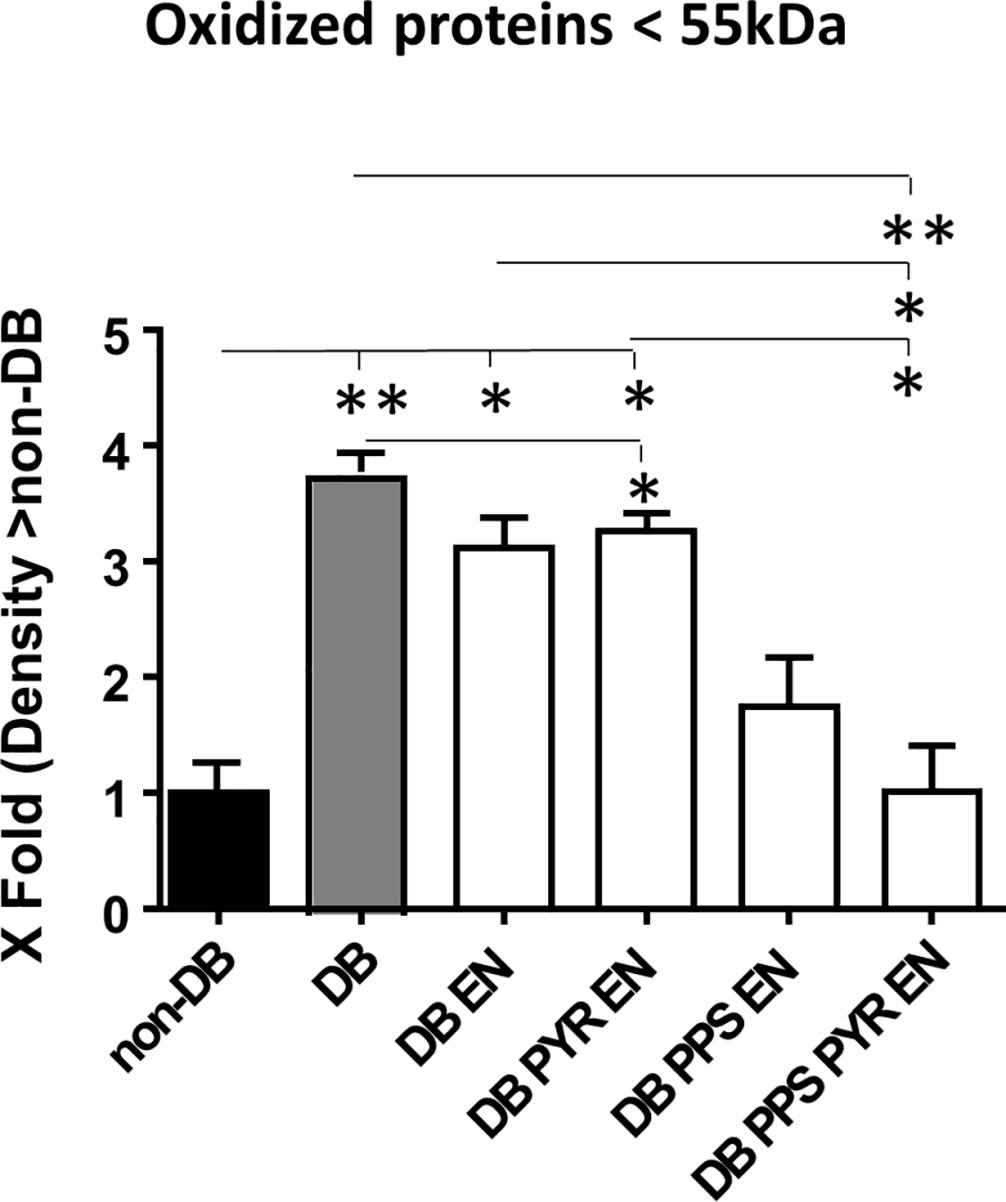
Oxidized kidney proteins < 55kDa, densitometric analyses. *p<0.05; **p<0.01. DB, diabetics; EN, enalapril; PPS, pentosan polysulfate; PYR, pyridoxamine.

The levels of oxidized proteins with a molecular weight <55 kDa were significantly increased in DB kidneys (*p*<0.01) (**Fig 3**). PYR+EN and PPS+PYR+EN reduced the levels of these proteins in DB kidneys (*p*<0.05 and *p*<0.01 respectively), reaching levels similar to non-DB controls. The levels of oxidized kidney proteins with a MW >55Kd were not increased in DB, but were lower than DB in the PPS+PYR+EN group (*p*<0.05) (**S1 Fig**).

### Independent association of inflammation and oxidative stress-related parameters and the albumin/creatinine ratio (ACR)

In a multiple linear regression analysis with ACR as the dependent variable, sMG and sTNFR1 levels as well as TGF*β*1, *RAGE* and *MCP1* mRNA levels appeared as significant predictors of ACR (all *p*<0.05) (**Table 2**). However, there was no independent link of ACR with sCML, iCML, iMG levels and AGER1 protein levels, and *Nrf2* and *ER*α mRNA levels. ACR levels were reduced to values comparable to non-DB controls only by PPS+PYR+EN (*p*<0.01). There were no associations with any of the other analyzed parameters when GS was used as the dependent variable.

**Table 2:** Multiple linear regression coefficients* to predict ACR.

Variables	Unstandardized Coefficients		Standardized Coefficients	*p* value
	*B*	*Standard error*	*Beta*	
**sMG**	0.164	0.066	0.306	0.019
**sTNFR1**	3.18 x 10^−4^	9.21 x 10^−5^	0.322	0.007
***TGFβ1* mRNA**	0.038	0.016	0.277	0.036
***RAGE* mRNA**	0.153	0.047	0.449	0.008
***MCP1* mRNA**	0.222	0.048	0.517	0.001

Dependent Variable: ACR (units) Predictive variables tested by backward method: blood glucose (mg/dl), sCML (U/mL), sMG (nmol/mL), iCML (U/mg protein), iMG (nmol/mg protein), sTNFR1 (pg/mL), AGER1 and SIRT1 protein levels, and *TGFβ1*, *MCP1*, *RAGE*, *Nrf2 a*nd *ERα* mRNA levels.

*(Constant) = 0.333. (R) = 0.945.

## Discussion

These results show that progressive GS in adult sclerosis-prone mice with controlled hyperglycemia can be reversed using a combination of FDA-approved drugs that are safe, readily available and inexpensive. Pentosan polysulfate+pyridoxamine+enalapril (PPS+PYR+EN) treatment: 1) reduced established GS and albuminuria; 2) increased anti-OS/Infl defenses and decreased pro-OS/Infl components, and 3) reduced elements of the sclerosis-prone phenotype in non-DB controls. Other combinations of these drugs had less marked results, suggesting that effective control of DKD may require addressing both extracellular and intracellular OS/Infl.

PPS and PYR are extensively utilized in the clinics and have good a safety profile. PPS is a hyper-sulfated, natural heparinoid with minimal anticoagulant effect (1/15^th^ that of heparin), and has significant anti-inflammatory properties [37]. It reduces CKD in several experimental mouse and rat models [22, 31, 38], and reduces inflammation and/or sclerosis in vascular grafts in animals and humans [26, 39]. PYR is a biguanide that inhibits intracellular OS/Infl by scavenging carbonyl compounds and hydroxyl radicals, that have been shown to reduce cellular anti-OS/Infl defenses [21]. PYR decreases intracellular OS/Infl in part by binding intracellular methylglyoxal (an AGE precursor), which is a very active oxidant formed during normal metabolism, but in excess amounts in diabetes [16, 17, 21, 40]. PYR has be shown to reduce DKD in animal models [40–42].

EN, an established standard in the clinical care for DKD with albuminuria [43]. When given as a single agent, it reduced GS and albuminuria less significantly than did PPS+PYR+EN. EN as a single drug or in combination with PPS or PYR significantly reduced *RAGE* below DB and non-DB levels. These data suggest that the RAAS system may be involved in the sclerosis-prone phenotype of ROP mice [44].

The fact that PPS+PYR+EN reduced GS and albuminuria below those in non-treated control ROP/Os+ controls provides critical support for a role for OS/Infl in the development of GS in mice and, perhaps, with the sclerosis-prone phenotype. Further support for the hypothesis that increased OS/Infl is a significant component of the development of GS is provided by two related studies. First, pre-anestrus hyperglycemic C57Bl6 mice have robust OS/Infl defenses and do not develop significant glomerular changes [2]. However, hyperglycemia induces a rapidly progressive inflammatory glomerular disease after menopause [9], a time at which OS/Infl defenses, including ER, have been compromised [22, 24, 25, 45, 46]. Secondly, C57Bl6 mice transgenic for SV40Tag, which induces inflammation, develop progressive glomerulosclerosis at a young age [47]. Thus, intact OS/Infl defenses may play a role in the resistance to glomerulosclerosis and CKD in mice. Finally, impaired OS/Infl defenses are part of the sclerosis-prone phenotype in mice.

SIRT1, a key member of the OS/Infl defense system, may protect against GS by suppressing downstream TGFβ1 signaling [48, 49]. *Sirt1* mRNA levels are reduced in DB and aged C57Bl6 mice [50] and humans [8]. The current study provides evidence that SIRT1, while it is reduced in hyperglycemic ROP Os/+ mice, their levels can be increased with drugs. Since SIRT1 levels in the PPS+PYR+EN group exceeded those in the non-treated litter-mate controls (non-DB), suggests that reduced SIRT1 levels may be an important part of the sclerosis phenotype and that intact SIRT1 levels may provide protect against hyperglycemia-induced GS. Finally, it also suggests that intact SIRT1 levels may modify the sclerosis-prone phenotype.

AGER1, another important member of the anti-oxidant defense system [51], is present both on the cell surface where it serves as an AGE receptor, and in the endoplasmic reticulum, where it is a part of the oligosaccharide transferase complex (OST) [52]. Cell surface AGER1 acts in synergism with SIRT1. While AGER1 protein levels were decreased in DB, all treatments except EN increased AGER1 to non-DB levels. This may have contributed to decreased OS/Infl and GS.

*Nrf2* and *ERα* mRNA levels, both of which are redox-sensitive transcription factors, were reduced in both non-DB and DB ROP OS/+ mice. Nrf2 is an essential component in the transactivation of genes containing antioxidant-response elements in their regulatory regions, which allows the coordinated expression of genes associated with protection against OS/Infl [53, 54]. PPS+PYR+EN enhanced the expression of *Nrf2* in the DB kidney to levels above those in non-DB. *ERα* mRNA levels were markedly suppressed in DB and while they were increased by EN+PYR, they were restored to non-DB levels only by EN+PYR+PPS. The potential clinical relevance of this finding is that reduced ER function in diabetic patients with CKD was restored by reducing inflammation [55–57]. Thus, decreased OS/Infl defenses, a component of the sclerosis-prone phenotype, appear to be modifiable.

The multiple linear regression analysis corroborated the contribution of OS/Infl factors (sMG and sTNFR1 levels and *TGFβ1*, *RAGE* and *MCP1* mRNA levels) as being contributing factors in the pathogenesis of albuminuria. It also suggests that these factors might prove useful in assessing the utility of interventions in early DKD.

This pilot study has several limitations including the relatively short follow-up period, the absence of a treated non-DB control or male mice, the presence of tissue and OS/Infl marker studies only at study end, and the small size of the study groups. Since the relatively large amount of oxidant AGEs in the usual animal chow, was not modified in this study [17, 18], a more pronounced effect of dietary oxidants on OS/Infl might have been seen if their restriction, by either low AGE food or drugs that bind AGEs in food had been utilized [55, 56]. These pilot data suggest that additional drugs that might preserve OS/Infl defenses should be explored.

In summary, the present pilot study in hyperglycemic and naive ROP/Os+ mice demonstrates that control of OS/Infl with currently available and relatively inexpensive and safe agents can modulate established GS, and DKD, decrease the sclerosis-prone phenotype in mice. The relevance of these data to humans with progressive DKD and a detailed study of the mechanisms involved, or other types of genetically determined CKD remains to be determined.

## Supporting information

S1 Fig Evaluation of the pool of oxidized kidney proteins. (PPTX)
