# Supplementary material for: Pharmacologic control of oxidative stress and inflammation determines whether diabetic glomerulosclerosis progresses or decreases: A pilot study in sclerosis-prone mice
Source: PLoS One. 2018 Sep 25;13(9):e0204366. doi: 10.1371/journal.pone.0204366 (PMC6155507; doi:10.1371/journal.pone.0204366)
Supplement: S1 Fig — (PPTX) [file pone.0204366.s001.pptx]

## Slide 1
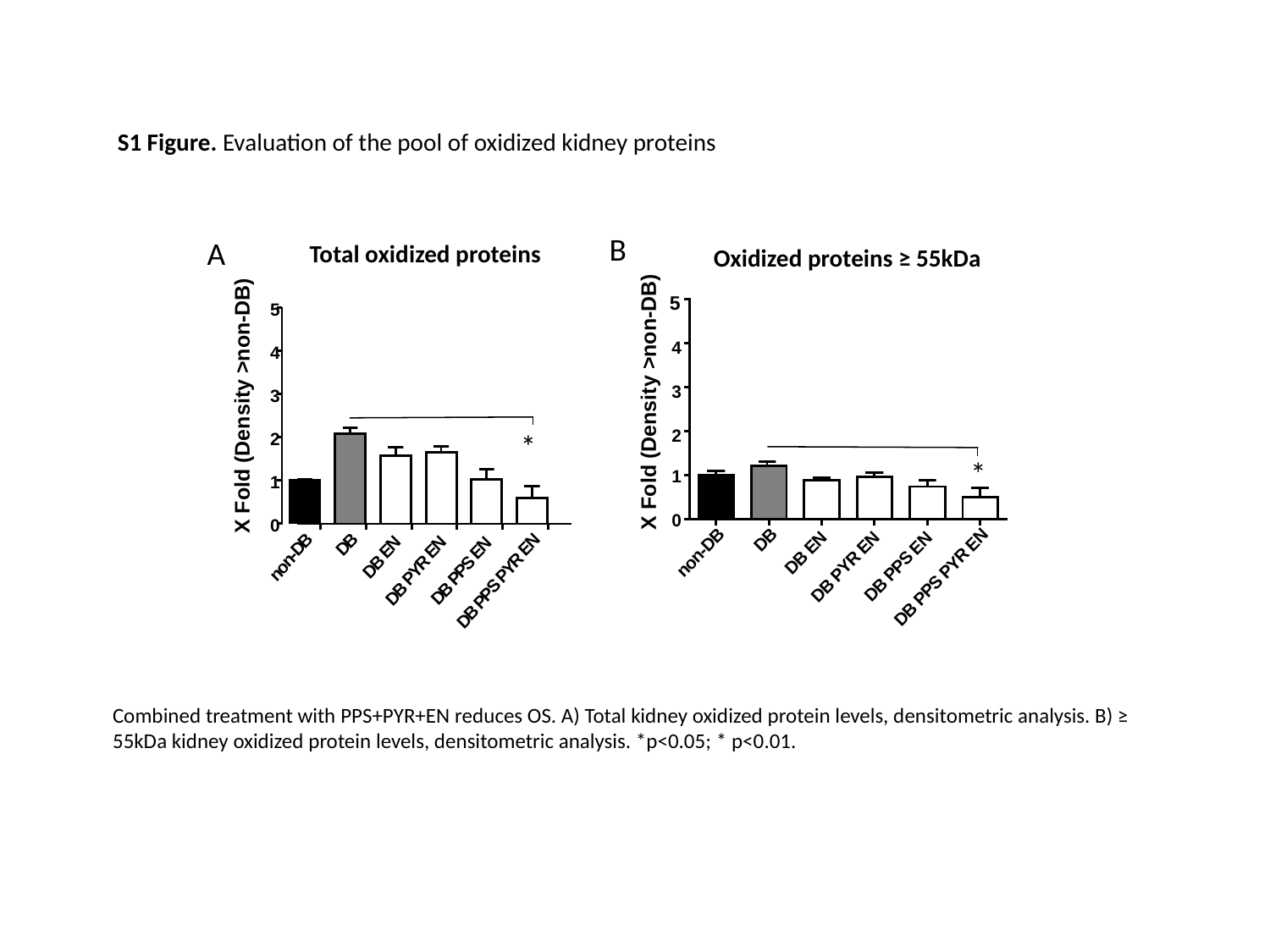

S1 Figure. Evaluation of the pool of oxidized kidney proteins
B
A
 Total oxidized proteins
Oxidized proteins ≥ 55kDa
5
4
3
*
2
1
0
B
B
N
N
N
N
E
D
D
E
E
E
-
R
n
S
B
R
o
Y
P
Y
D
n
P
P
P
S
B
B
P
D
D
P
B
D
X Fold (Density >non-DB)
5
4
3
2
*
1
0
B
B
N
N
N
N
D
E
D
E
E
E
-
R
n
S
B
R
o
Y
P
Y
D
n
P
P
P
S
B
B
P
D
D
P
B
D
X Fold (Density >non-DB)
Combined treatment with PPS+PYR+EN reduces OS. A) Total kidney oxidized protein levels, densitometric analysis. B) ≥ 55kDa kidney oxidized protein levels, densitometric analysis. *p<0.05; * p<0.01.
